# Estimating Abundances of Interacting Species Using Morphological Traits, Foraging Guilds, and Habitat

**DOI:** 10.1371/journal.pone.0094323

**Published:** 2014-04-11

**Authors:** Robert M. Dorazio, Edward F. Connor

**Affiliations:** 1 U.S. Geological Survey, Southeast Ecological Science Center, Gainesville, Florida, United States of America; 2 Department of Biology, San Francisco State University, San Francisco, California, United States of America; Wildlife Conservation Society, India

## Abstract

We developed a statistical model to estimate the abundances of potentially interacting species encountered while conducting point-count surveys at a set of ecologically relevant locations – as in a metacommunity of species. In the model we assume that abundances of species with similar traits (e.g., body size) are potentially correlated and that these correlations, when present, may exist among all species or only among functionally related species (such as members of the same foraging guild). We also assume that species-specific abundances vary among locations owing to systematic and stochastic sources of heterogeneity. For example, if abundances differ among locations due to differences in habitat, then measures of habitat may be included in the model as covariates. Naturally, the quantitative effects of these covariates are assumed to differ among species. Our model also accounts for the effects of detectability on the observed counts of each species. This aspect of the model is especially important for rare or uncommon species that may be difficult to detect in community-level surveys. Estimating the detectability of each species requires sampling locations to be surveyed repeatedly using different observers or different visits of a single observer. As an illustration, we fitted models to species-specific counts of birds obtained while sampling an avian community during the breeding season. In the analysis we examined whether species abundances appeared to be correlated due to similarities in morphological measures (body mass, beak length, tarsus length, wing length, tail length) and whether these correlations existed among all species or only among species of the same foraging guild. We also used the model to estimate the effects of forested area on species abundances and the effects of sound power output (as measured by body size) on species detection probabilities.

## Introduction

Much of ecological research is driven by the desire to understand observed patterns of variation in the abundance or occurrence of individual plants or animals. While this variation may be associated with the environmental requirements, dispersal ability, and biotic interactions of each species, the relative importance of these components can be difficult to quantify or assess. Part of the difficulty is methodological – that is, induced by limitations or deficiencies in the methods that are used to analyze data from community-level surveys.

For example, inferences about species interactions or associations traditionally have focused on examining patterns of co-occurrence between pairs of species observed at several locations [1]. Such data have been examined using so-called “null model” analyses aimed primarily at testing hypotheses about the importance of competitive interactions within a community of species [2]–[4]. In these analyses species co-occurrences are often quantified as a function of a community's incidence matrix [5], [6], which includes the binary occupancy state (presence or absence) of each species at each sample location. More recently, parametric statistical models have been developed to estimate the effects of one species on another. In some of these models the effects of imperfect detectability of a species are ignored [7], [8]; in others the errors in detection of individuals are explicitly accounted for in the model's underlying assumptions [9]–[12]. An advantage of using parametric modeling is that the strengths of interspecific interactions are specified in terms of estimable model parameters, allowing the null hypothesis (no interactions) to be tested and specific alternatives to be quantified.

While these recent advances are useful, species occupancy state provides a relatively coarse summary of a species' local population size 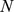 – specifically that 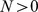 (species is present) or 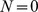 (species is absent). Interactions between species are likely to be inferred more easily by analyzing a matrix of species- and location-specific abundances. For example, the abundances of strongly interacting species are likely to be positively or negatively correlated; however, these correlations may be difficult to detect if analyses are limited to frequencies of co-occurrence between species. Furthermore, analyses of incidence matrices may be more sensitive to sampling errors because a species of low abundance may incorrectly be regarded as absent even though 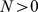 for this species. For these reasons, models of species counts are expected to be more useful than models of species occurrences in estimating the magnitude of interspecific interactions.

Several approaches have been used to analyze species-specific counts of individuals (plant or animals) encountered while surveying a set of ecologically relevant locations. Many of these approaches assume pairwise independence among species and focus on examining the effects of environmental or habitat covariates on species abundance. In this approach the counts of individuals of different species are aggregated to obtain a total count of individuals that belong to a community, a guild, or a functional group [13]–[19]. Linear models are used to relate these totals to location-specific measurements of habitat. While statistical analyses of such aggregated counts obviously suffer from a loss of information, the results of the analyses also can be misleading. For example, estimates of covariate effects on abundance may primarily reflect the effects on the most abundant species. The counts of rare and potentially strongly interacting species will be swamped by the counts of the abundant species. Also, significant effects of covariates on abundance may be difficult to detect if species of nearly equal abundance have opposite responses to the same covariates.

Another approach often used in the analysis of species-specific counts involves multivariate ordination [18], [20]–[22]. In these analyses the distance-based metric selected for ordination generally fails to specify the mean-variance relationship in the counts correctly, leading to conclusions (such as statistical significance of covariate effects) that confound differences in mean with differences in variance [23].

In other studies species-specific counts are analyzed to detect potential associations between species that may suggest the presence of competitive interactions or habitat associations. Not surprisingly, some of these analyses are based on null models for testing pairwise interactions between species [24]–[27]. These analyses differ in underlying assumptions and in the indices used to summarize patterns of species co-occurrence and spatial aggregation. Importantly, however, rejection of the null hypothesis (no interactions) is not accompanied by estimation of the magnitude of interspecific interactions – primarily because null model analyses usually are conducted nonparametrically (see [28] for an exception).

Another approach to the analysis of species-specific counts is based on univariate, linear-regression models wherein counts of one species are regressed on habitat measurements and on standardized counts of other species to estimate pairwise species interaction strengths (“competition coefficients”) [29]–[31]. In this approach counts of individuals are regarded as surrogates of abundance; however, these regression models do not honor the discrete nature of the counts (which are assumed to be normally distributed), and the counts are treated as both stochastic response variables and fixed predictors, even though both are clearly outcomes of sampling.

An alternative approach is based on fitting statistical models that assume the counts of individuals are distributed as mixtures of Poisson and lognormal distributions. Similar to the regression models, the counts are regarded as surrogates of abundance; however, Poisson-lognormal mixtures account for discreteness of the counts and for the increase in variance with mean that is induced when individuals are distributed randomly or in spatial aggregations [32], [33]. A univariate Poisson-lognormal mixture for modeling species-specific counts of individuals observed at a single location was proposed by [34]. This model was extended by [35] for pairs of locations but considered only stochastic sources of variation in abundance among locations (as opposed to specifying the effects of differences in habitat on species abundances). In both models species-specific abundances were assumed to vary exchangeably among species (via the lognormal distribution) and the abundances of different species were not assumed to be correlated (say, as a consequence of interactions among species). The multivariate Poisson-lognormal distribution [36] was developed to allow abundances of different species to be correlated; however, heterogeneity in species abundances among locations was not specified as a function of habitat, which may explain why the model has not attracted much attention by ecologists. This model was extended recently to include the effects of location-specific covariates on species abundances [37].

An implicit assumption of all of the count models described above is that each observed count is assumed to equal the actual abundance of individuals. In other words, every individual of every species is assumed to be detected with the same probability (one) at every sample location. This assumption is seldom satisfied in surveys of natural communities because individuals which are present and available for detection are routinely missed during sampling [38], [39]. Furthermore, the probability of detection generally varies considerably among species. When an entire community is sampled, many species may be represented by only one or a few individuals at each location, so it is crucial that analyses account for the effects of imperfect detectability if species abundances are to be estimated accurately [40], [41].


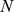
-mixture models [42] allow abundances to be estimated from samples of repeated point counts while accounting for imperfect detections of individuals. In these models the number of individuals detected during a survey (i.e., the observed count) is assumed to equal the sum of 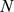 independent Bernoulli outcomes each with identical success (detection) probability. However, since the abundance of animals at each sample location is unknown, 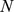 must be estimated by coupling the Bernoulli model of the observations with a model that specifies how 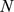 varies among locations. This model of abundance includes a mixing distribution for 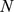, and hence the name 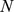-mixture models. Although extensions of these models have been proposed for multiple species [43]–[45], to our knowledge [46] provide the only published study in which 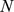-mixtures have been used to analyze counts from multiple, interacting species. In this study, though, the effects of habitat on species abundances were not included in the analysis.

Our study was motivated by a desire to determine whether the effects of environmental (habitat) covariates or interactions between species were more influential in determining the abundances of individual species. Specifically, we sought to develop a statistical model of counts from community-level surveys that overcomes many of the limitations of existing models. In the paper we propose a model to estimate the abundances of potentially interacting species using repeated point counts of each species observed while sampling a set of ecologically relevant locations. This model includes two extensions of the multivariate Poisson-lognormal mixture: it allows abundances of species with similar traits to be correlated and it allows the effects of habitat on abundance to be estimated. Applying the 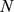-mixture idea, we combine this model of species abundances with a binomial model of the observed counts that allows the probability of detection to be estimated for each species in conjunction with its abundance. To illustrate the potential benefits of this approach, we analyzed point counts obtained while sampling a community of forest birds during the breeding season. These data are far from ideal for our analysis, but they are typical of the kind of information obtained in avian community-level surveys. Our objective here is to provide a proof-of-concept of our statistical approach, not an exhaustive analysis of the avian point count data.

## Statistical Analysis

In this section we describe a statistical model for estimating the abundances of potentially interacting species encountered while surveying a set of distinct locations. The sample locations are assumed to be representative of some collection of locations that is considered to be ecologically relevant – as in a metacommunity of species. In the model we assume that abundances of species with similar traits are potentially correlated and that these correlations, when present, may exist among all species or only among functionally related species (such as members of the same foraging guild). We also assume that abundances vary among locations owing to systematic and stochastic sources of heterogeneity. For example, if abundances differ among locations due to differences in habitat, then measures of habitat can be included in the model as covariates. Naturally, the quantitative effects of these covariates are assumed to differ among species.

Our model also accounts for the effects of detectability on the observed counts of each species. This aspect of the model is especially important for rare or uncommon species that may be difficult to detect in community-level surveys. Estimating each species' detectability requires some sampling locations to be surveyed repeatedly using different observers or different visits of a single observer. These within-location replicates provide the information needed to estimate both abundance and detectability of each species.

### Modeling species abundances

We begin by describing a model of species- and location-specific abundances, which are not directly observable but are the quantities of primary scientific interest. Let 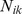 denote a random variable for the number of individuals of species 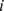 that are present and available to be observed at sample location 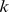 (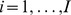; 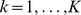). 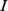 is the number of distinct species observed among all 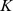 sample locations. We assume that the expected abundance of individuals of species 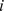 at location 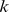, say 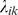, is constant during the surveys of each location and that 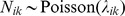.

We specify location-specific differences in the abundance of individuals using a log-linear regression model of 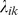: 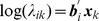. (A prime symbol indicates the transpose of a vector or matrix.) In this model the vector 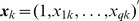 includes the observed values of 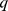 covariates thought to be informative of abundance at location 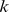. For example, 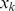 may include measures of habitat at location 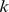. The parameter vector 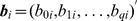 includes an intercept and the effects of the 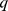 covariates. If the values of each covariate are centered at zero, the intercept parameter 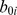 denotes the log-scale, expected abundance of species 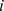 at the average value of the covariates.

To allow for potential correlations in abundance among species, we specify dependence among the 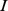 intercepts using the multivariate normal distribution:
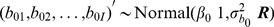
(1)where 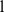 is an 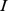-vector of ones, 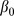 and 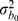 parameterize the mean and variance among intercepts, and 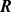 is their 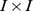 correlation matrix. This specification follows the approach taken by [36] but without an unstructured correlation matrix. To ensure that 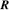 is positive definite, we borrow an idea from geostatistical modeling [47], [48] and use an exponential function, 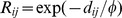, to specify the correlation between abundances of species 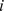 and 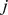 as a function of a positive, scalar-valued parameter 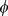 and a measure of dissimilarity 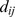 in traits of species 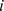 and 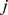. For simplicity, we propose the Euclidean distance 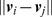 between trait vectors 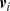 and 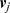 as a measure of 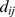, but alternative measures of dissimilarity are possible (provided 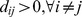). Thus, if traits of species 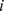 and 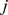 are similar (low 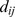), their abundances are positively correlated under this model.

Our decision to specify correlations in abundance as a function of similarity of morphological traits was motivated by the need to ensure that 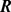 is positive definite; however, the idea that species of similar morphology may have positively correlated abundances also has ecological support. For example, two partially opposing views exist about how species interactions might affect correlations in abundance between species. One view is based on the concept of limiting similarity in which species of similar morphology compete for the same resources [49], and coexistence of these species requires them to “partition” the environment to minimize overlap in resource use [50]. Under this view one might expect that species of similar morphology – and therefore similar resource use – might not co-occur, or if they do co-occur, their abundances might be negatively correlated. However, the spatial scale at which resource partitioning occurs is not known. The shared-niche model suggests that at larger spatial scales the occurrences and abundances of competing species may be positively correlated even though resources are partitioned at a smaller scale [51]. In addition, most species of birds do not maintain interspecific territories. Finally, [52]'s classic study of warblers suggests that resource partitioning among avian species – if it occurs – might be at a very fine scale (e.g., different parts of the forest canopy). Therefore, estimated abundances of individuals vulnerable to detection in a point-count survey may not reflect competitively mediated resource partitioning because the region of detection may – and often does – include a wide variety of microhabitats and individual species territories.

Another view is based on the idea that species which are morphologically similar and consume the same resources are likely to aggregate at the same sites where shared resources or habitat is abundant. There is evidence that closely related species, which tend to be morphologically similar, tend to co-occur more than expected [53], [54] or to have their occurrences or abundances positively correlated [8], [55], at least at larger spatial scales. Species co-occurrence and positive correlations in abundance probably reflect associations caused by shared habitat preferences or shared resource requirements. In light of these two views, we suggest that at the scale of our point-count surveys the decision to specify correlations in abundance between species as a positive function of their morphological similarity is not unreasonable.

To complete the model of species abundances, we assume that species-specific effects of the 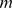
th covariate of mean abundance (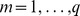) vary exchangeably among species as follows:
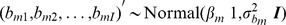
where 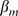 and 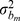 are mean and variance parameters, respectively, and 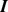 is a 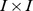 identity matrix. Thus, while the effects of any particular habitat covariate may differ among species, these effects are assumed to vary independently among species. In addition, we assume mutual independence between the vector of intercept parameters and the vectors of covariate effects.

#### Submodels of species abundances

Two special cases of our abundance model are noteworthy. First, we may consider a model wherein correlations in abundance are assumed only among related species. For example, we may assume that abundances are positively correlated among species of the same foraging guild but not correlated with species in different guilds. If the species are ordered by guild, this model is essentially the same as that specified in Eq. 1 except that the correlation matrix 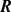 is block-diagonal:
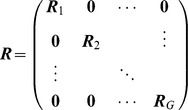
where each block corresponds to the correlation matrix of a distinct foraging guild 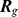 (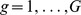). Elements of each submatrix 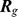 depend on the traits of species in guild 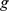 and on the parameter 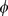 as described earlier.

Another special case of our abundance model corresponds to absence of correlations in abundance wherein 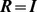. In this model we assume that all species are ecologically similar so that their intercepts (i.e., log-scale mean abundances) are exchangeable. In this case differences in species traits are assumed to be uninformative of differences in species abundances, and the abundance of one species is assumed to be independent of the abundance of another species.

### Modeling species counts

Species- and location-specific abundances usually cannot be observed directly in multi-species surveys. Instead, counts of individuals are typically observed, but the counts are subject to errors in detection of individuals. An observed count generally underrepresents the number of individuals of each species present, and this bias is particularly true for uncommon species that are easily missed. Therefore, a model is needed to relate the observed counts of each species to the actual abundance of that species.

We assume that 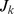 independent surveys are conducted at the 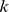
th sample location such that each species' abundance remains constant during the period of sampling. These surveys may be conducted by independent observers or by repeated visits of a single observer. As proposed by [42], the idea is to treat the individuals at each site as a closed population of size 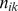 (a realization of the random variable 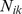) and to use the replicate counts of these individuals to estimate the probability of detection per individual.

Given these assumptions, we describe a model of the observed counts that allows detection probabilities to be estimated for each species. Let 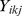 denote a random variable for the number of individuals of species 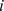 detected during the 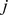
th survey of sample location 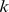 (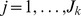). We assume that 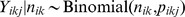, where 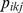 denotes the conditional probability of detecting an individual of species 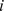 during the 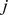
th survey of site 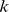. In addition, because surveys are conducted independently at each site, we assume that each site's observations are conditionally independent, that is,
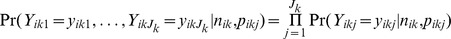



Various models of 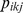 may be constructed depending on the availability of species- or site-specific covariates thought to be informative of detectability. For example, suppose a vector of 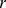 covariate measurements 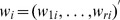 is thought to be informative of the detectability of species 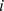. The effects of these covariates on 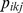 can be specified using a logit-linear regression model with random intercepts as follows:
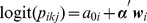


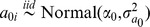
In this formulation latent (unobserved) sources of heterogeneity in detection among species are specified exchangeably using the normal distribution, whereas systematic (observed) sources of heterogeneity in detection are specified by the regression parameters 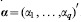. We used this example because it corresponds to the model used in our analysis of point counts of avian species. More generally, however, the observation model – that is, the model of the observed counts – depends on the availability of auxiliary data, which could include species-, location- or even survey-specific covariates. Therefore, this approach to modeling building is extremely versatile. Adopting a hierarchical approach to model building [41], wherein separate models are used to describe the ecological and sampling processes, allows us to specify an explicit relationship between the observed counts and the latent abundances of each species.

### Estimating model parameters

The hierarchical model described in the previous sections would be difficult to fit using classical methods owing to the high-dimensional and analytically intractable integrations involved in evaluating a marginal (integrated) likelihood function of upper-level parameters (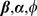 and variance parameters). We therefore adopted a Bayesian approach to inference and used Markov chain Monte Carlo methods [56] to fit the model and to estimate its parameters. In Appendix S1 we describe the algorithm used to calculate summaries of the posterior distribution and other ecologically relevant functionals of the Markov chain.

We used a posterior-predictive loss criterion [57] to compare the three models of species-specific abundances. Specifically, we used the following criterion:
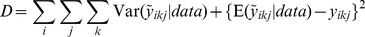
which minimizes the expected squared-error loss between the observed counts (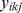) and the counts predicted under a model (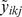). Models with lower values of this criterion are preferred because they have lower predictive variance (first term in 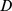) and lower lack of fit (second term in 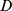).

## Description of Data Sets

We analyzed species- and location-specific counts of 73 avian species observed while sampling 46 tracts of forest in southeastern Connecticut [58]. Tracts were defined as areas of forest not interrupted by powerlines, highways, or unforested areas wider than 10 m. [58] provide a detailed description of the species, sampling methods, and environmental covariates. Briefly, birds were detected aurally (and sometimes visually) within each of 89 sample locations during three 20-min, point-count surveys (100 m radius). During each survey the locations of birds detected were mapped, and birds that were spatially separated and singing were treated as distinct individuals. Repeated surveys of the same location were conducted during early morning hours (530 h to 1000 h) on three separate days during the breeding season (21 May to 11 July). The sample locations were sufficiently far apart that birds were unlikely to have been observed at more than one location. The movements of birds were limited during the sampling period owing to mating and nesting behaviors.

Of the various environmental covariates measured by [58], tract-level measures of forested area appeared to be the best predictors of counts of avian species. We therefore used one of these measures – forested area (ha) within 2 km of a tract's center – to describe differences in avian habitat among sample locations and to predict the abundance of individuals of each species at these locations. Appendix S2 contains the species-specific point counts and measurements of forested area at each sample location.

[58] also used habitat to classify each species into one of three categories: (1) interior species, whose territories are normally restricted to the interior of a forest, (2) edge species, whose territories are primarily concentrated on a forest's edge, and (3) interior-edge species, whose territories may include both forest interior and edge. We did not use these categories to model species abundances; however, we did examine whether our model-based estimates of species-specific abundances appeared to agree with [58]'s classification of species.

We used several kinds of information about individual species to inform different components of our model. For example, we used body mass data [59] to estimate the sound power output (mW) of singing males according to an allometric relationship reported by [60]. We reasoned that birds with greater sound power output (i.e., higher body mass) would, on average, be more detectable than birds with lower sound power output.

We also used species-specific morphological information to model correlations in abundance between species. We obtained measures of morphological traits (body mass, beak length, tarsus length, wing length, and tail length) for each species (primarily from [61]). We then reduced the dimensionality of the trait measurements by performing a principal components analysis on their correlation matrix and by computing principal-component scores from the two eigenvectors associated with the highest two eigenvalues.

We examined whether abundances of all species or only functionally related species were correlated by using foraging guilds to define inter-relatedness among species. We used foraging guilds assigned by [62] for the breeding season if a species used different foraging strategies seasonally; if not, we used the year-round guild assignment of a species. Appendix S3 contains a species list with guild assignments, morphological traits, and sound power estimates.

We fitted three models to the avian point counts. Each model included the effects of forested area on species-specific abundances and the effects of sound power output on species-specific detection probabilities. The three models differed in the assumed pattern of correlation between abundances of different species – that is, we assumed no correlation, correlation among species of the same foraging guild, or correlation among all species.

## Results

**Figure 1:**
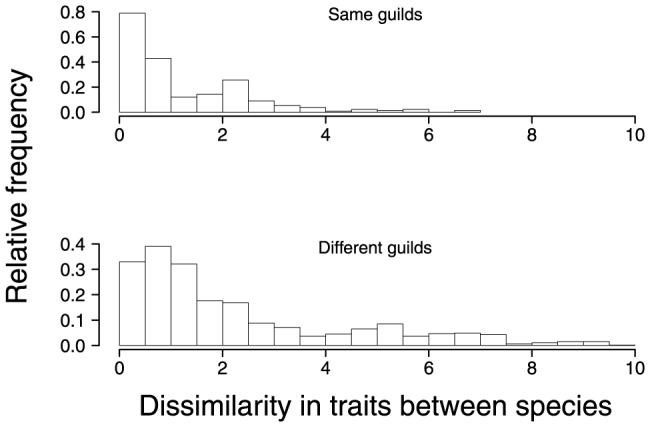
Dissimilarity in morphological traits between species of same foraging guild (upper panel) and between species of different foraging guilds (lower panel).

**Figure 2:**
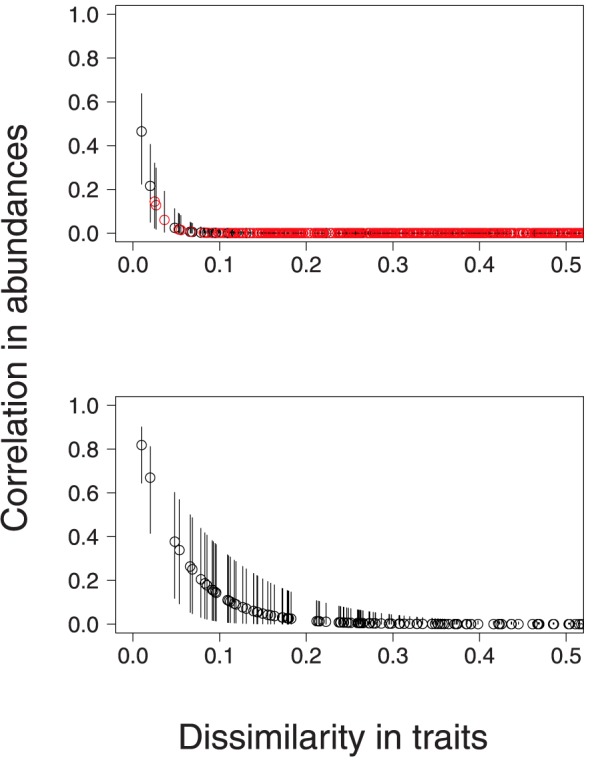
Estimated correlations in abundance between species (posterior medians and 95% credible intervals) as a function of dissimilarity in their morphological traits. Black indicates species of same foraging guild; red indicates species of different foraging guilds. Upper panel corresponds to estimates obtained by assuming correlated abundances among all species; lower panel correponds to estimates obtained by assuming correlated abundances among species of same foraging guild. Estimated correlations are equal to zero for species with trait dissimilarities greater than 0.5.

Based on our model-selection criterion, the model in which abundances were assumed to be correlated among all species was favored over the other, less-complex models, but the differences between models were not that great (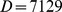 (correlated abundances among all species), 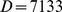 (uncorrelated abundances), 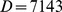 (correlated abundances among species of the same foraging guild)). The similarity in these model fits was evident also in estimates of their parameters (Table 1). For example, estimates of 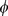, which determine the magnitude of correlations in abundance, were close to zero even though the dissimilarities in morphological traits ranged from 0 to 10 (Figure 1). This result was evident also in the estimates of correlation between abundances of different species. Abundances of species of very similar morphology were positively correlated; however, the abundances of most species were not strongly correlated (Figure 2).

**Table 1:** Posterior means and 95% credible intervals for the parameters of three models.

	Model 1			Model 2			Model 3		
Parameter	Mean	2.5%	97.5%	Mean	2.5%	97.5%	Mean	2.5%	97.5%
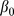	−0.741	−1.165	−0.302	−0.786	−1.270	−0.300	−0.774	−1.186	−0.381
	(0.008)	(0.008)	(0.008)	(0.009)	(0.012)	(0.009)	(0.007)	(0.008)	(0.007)
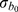	1.472	1.202	1.798	1.641	1.308	2.060	1.475	1.193	1.806
	(0.004)	(0.004)	(0.006)	(0.007)	(0.006)	(0.010)	(0.005)	(0.005)	(0.007)
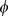	–	–	–	0.069	0.003	0.240	0.016	0.001	0.050
				(0.002)	(0.0005)	(0.006)	(0.0003)	(0.0001)	(0.001)
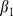	−0.112	−0.291	0.066	−0.112	−0.292	0.070	−0.108	−0.286	0.072
	(0.001)	(0.002)	(0.001)	(0.001)	(0.002)	(0.002)	(0.001)	(0.002)	(0.002)
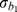	0.696	0.559	0.860	0.698	0.564	0.862	0.693	0.558	0.854
	(0.002)	(0.002)	(0.003)	(0.002)	(0.002)	(0.003)	(0.002)	(0.002)	(0.003)
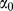	−1.696	−2.105	−1.399	−1.649	−2.019	−1.331	−1.637	−1.955	−1.347
	(0.009)	(0.012)	(0.009)	(0.009)	(0.011)	(0.009)	(0.007)	(0.010)	(0.007)
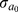	0.883	0.620	1.242	0.849	0.612	1.161	0.835	0.595	1.133
	(0.008)	(0.007)	(0.010)	(0.007)	(0.006)	(0.009)	(0.007)	(0.006)	(0.008)
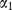	−0.669	−1.031	−0.288	−0.631	−1.036	−0.170	−0.631	−1.002	−0.283
	(0.011)	(0.011)	(0.011)	(0.013)	(0.013)	(0.013)	(0.010)	(0.011)	(0.011)

Model 1 assumes uncorrelated abundances. Model 2 assumes correlated abundances among species of the same foraging guild. Model 3 assumes correlated abundances among all species. Monte Carlo standard errors are given in parentheses.

**Figure 3:**
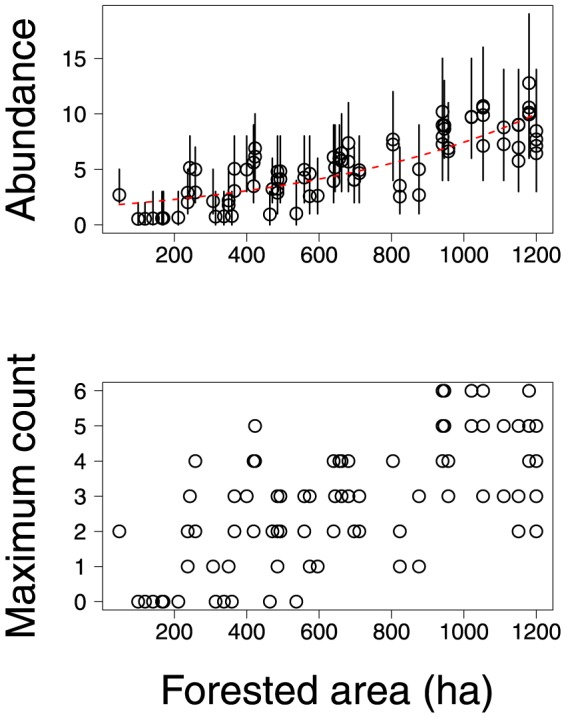
Maximum observed count of ovenbirds and estimates of their abundance (posterior means and 95% credible intervals) at sample locations. Dashed line indicates the estimated relationship between mean abundance of ovenbirds and forested area.

**Figure 4:**
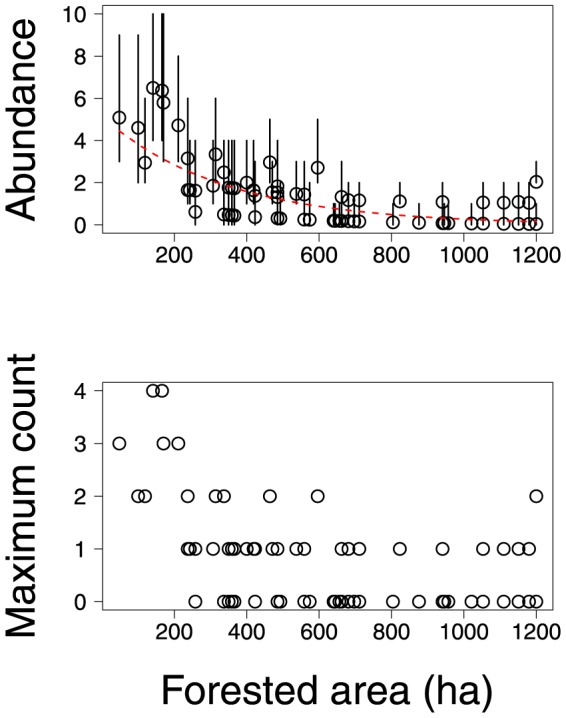
Maximum observed count of catbirds and estimates of their abundance (posterior means and 95% credible intervals) at sample locations. Dashed line indicates the estimated relationship between mean abundance of catbirds and forested area.

In contrast to morphological traits, habitat – as measured by forested area – appeared to have a strong influence on avian abundances. The effects of forested area were significantly positive for 15 species and significantly negative for 23 species (Appendix S4). As an illustration Figure 3 shows that estimates of ovenbird abundance were generally higher at locations with greater forested area. This trend was evident also in the maximum counts of ovenbirds detected at sample locations. Estimates of catbird abundance provide an example of the opposite trend, i.e., lower abundances and lower counts at sample locations with greater forested area (Figure 4).

**Figure 5:**
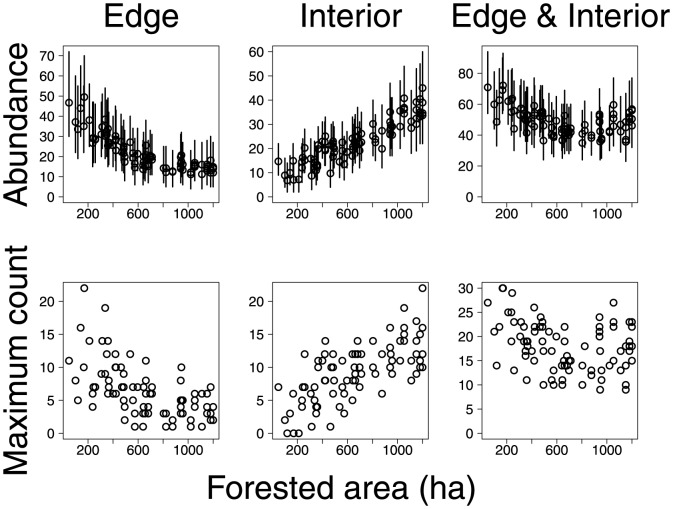
Maximum observed count of forest edge species, forest interior species, and forest edge and interior species and estimates of their abundance (posterior means and 95% credible intervals) at sample locations. Dashed line indicates the estimated relationship between mean abundance of birds and forested area.

Our estimates of abundances appear to agree with the classification of species proposed by [58]. The estimated abundances of forest interior species increased with the forested area of sample locations (Figure 5), whereas edge species were more abundant at sample locations with less forested area. The estimated abundances of interior-edge species were highest at sample locations with either low or high forested area, suggesting that this category may contain a mixture of species that prefer interior or edge habitats.

**Figure 6:**
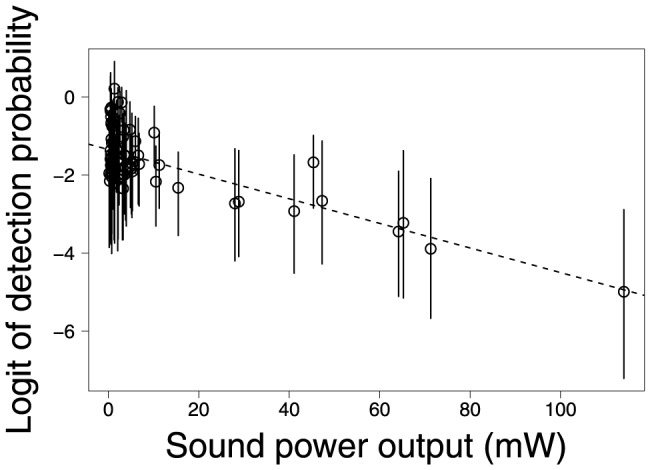
Estimated probability of detection (on logit scale with 95% credible interval) vs. sound power output (mW) of each species.

Contrary to our prior beliefs about detectability of birds, species-specific estimates of detection probability descreased with increases in sound power output (Figure 6); however, most of the decrease was associated with larger species. The estimated detection probabilities of smaller species were highly variable (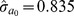). Although larger birds have higher sound power outputs, they also vocalize less frequently than smaller birds, and calls of larger birds tend to be lower in frequency and more easily masked by background noises [63]. A more complete model of heterogeneity in species-specific detection probabilities might include measures of average acoustic frequency and how often each species calls.

## Discussion

Our model extends the multivariate Poisson-lognormal mixture proposed by [36] to include effects of habitat covariates on abundance, to specify pairwise correlations in abundance between species in terms of morhological similarity, and to account for errors in detection of individuals. The model is a multispecies, 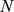-mixture model because the observed counts of each species are modeled conditional on a set of latent abundance parameters and a separate component of the model is used to specify heterogeneity in abundance of individuals among species and locations. In our model information from replicated surveys within locations is used to inform species detection probabilities. In this regard our approach is similar to that of [43], [44] and [45]; however, in those models the abundances of different species are not assumed to be correlated. Our approach also differs from that of [37], who extended the multivariate Poisson-lognormal mixture for the effects of habitat but did not formally address the impact of detection errors on estimates of abundance.

In our analysis of avian point counts, habitat exerted strong effects on the abundances of most species. These effects were evident in estimates of abundance of individual species (Figures 3 and 4) and in the estimated abundances of groups of species classified by [58] (Figure 5). An important benefit of our modeling approach is that we can estimate the residual correlations in abundance between species having accounted for the effects of habitat on abundances. In our analysis the abundances of different species did not appear to be strongly correlated unless these species were very similar morphologically (Figure 2).

However, an important limitation of our model is that species abundances can only be positively correlated owing to the assumed structure of the correlation matrix 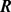. Ideally, we also would like to be able to estimate negative correlations (e.g., those induced by competitive interactions between species). For example, [36]'s multivariate Poisson-log normal mixture assumes
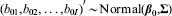
where 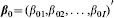 and 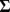 denotes an unstructured 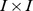 matrix of variances and covariances. Estimates of 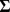 may include positive and negative covariances. Unfortunately, our attempts to fit this model to the avian counts were unsuccessful. We suspect that limited information in the data is responsible for some of the estimation problems. For example, in our model the species- and location-specific abundances 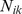 are latent parameters, not data. In the model of [36], 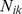 corresponds to an *observed* count, and the replicate observations among locations provide direct information about the parameters 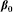 and 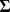. Also, the number of parameters to be estimated in 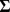 (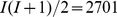) is relatively large compared to the number of sample locations (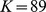); thus problems of parameter identifiability may have limited our ability to fit models with unstructured 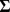.

This topic is obviously important and requires additional research. Being able to estimate correlations in abundance between species *and* the effects of environmental covariates on those species allows us to compare their relative magnitudes. However, a word of caution is in order here. While it may be tempting to interpret the correlations in abundance as evidence of interspecific interactions, the correlations also may be produced if the abundances of supposedly “interacting” species are influenced (positively or negatively) by unobserved – and therefore unmodeled – environmental covariates [64]. Care is therefore recommended during interpretation of results.

Despite the potential for misinterpretation, we believe that multispecies 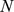-mixture models provide a useful conceptual framework for the analysis of community-level survey data. We anticipate that new data sets with greater numbers of sample locations and with alternative sampling protocols (e.g., double observers at the same location) will provide the information needed to estimate both positive and negative correlations in abundance between species. We also anticipate that these new data sets may allow species which are present but unobserved to be included in the model, as in multispecies occupancy models [65]. This extension would be useful for estimating species richness and other community-level measures of biodiversity [66].

## Supporting Information

Appendix S1 **MCMC algorithm used in model fitting.**
(PDF)

Appendix S2 **Species-specific point counts and measurements of forested area at each sample location (site).** Order of species (columns) is identical to order of rows in Appendix S3.
(CSV)

Appendix S3 **Morphological measurements, foraging guild, and sound power output of avian species.**
(PDF)

Appendix S4 **Estimates of model parameters for avian point-count data.**
(PDF)
