# Supplementary material for: Estimating Abundances of Interacting Species Using Morphological Traits, Foraging Guilds, and Habitat
Source: PLoS One. 2014 Apr 11;9(4):e94323. doi: 10.1371/journal.pone.0094323 (PMC3984154; doi:10.1371/journal.pone.0094323)
Supplement: Appendix S1 — MCMC algorithm used in model fitting. (PDF) [file pone.0094323.s001.pdf]

Estimating Abundances of Interacting Species Using Morphological Traits, Foraging Guilds, and Habitat

Robert M. Dorazio[1,*], Edward F. Connor[2]
1 U.S. Geological Survey, Southeast Ecological Science Center, Gainesville, Florida, USA
2 Department of Biology, San Francisco State University, San Francisco, California, USA
∗ E-mail: bdorazio@usgs.gov

Appendix S1: MCMC algorithm used in model fitting

To compute summaries of the posterior distribution, we used a Gibbs sampling algorithm [1] based on the following posterior density function:

$$[\boldsymbol{\theta}, \boldsymbol{a}_0, \boldsymbol{b}_0, \boldsymbol{b}_1, \boldsymbol{n} \mid \boldsymbol{Y}] = C\ [\boldsymbol{\theta}]\ [\boldsymbol{b}_0|\beta_0, \sigma_{b_0}, \phi] \prod_{i=1}^{I} [a_{0i}|\alpha_0, \sigma_{a_0}] \left\{ \prod_{m=1}^{q} [b_{mi}|\beta_m, \sigma_{b_m}] \right\} \prod_{k=1}^{K} [n_{ik}|b_{0i}, b_{1i}] \prod_{j=1}^{J_k} [y_{ikj}|n_{ik}, \boldsymbol{\alpha}, a_{0i}]$$

where C denotes the normalizing constant[1] of the posterior distribution and where $\boldsymbol{\theta} = (\boldsymbol{\alpha}, \boldsymbol{\beta}, \sigma_{a_0}, \sigma_{b_0}, \sigma_{b_1}, \phi,)'$. (Note that we specify probability density (and mass) functions using bracket notation wherein $[x, y]$ denotes the joint density of random variables X and Y, $[x|y]$ denotes the conditional density X given y, and $[x]$ denotes the unconditional (marginal) density of X.)

Assuming mutually independent priors for the parameters in $\boldsymbol{\theta}$ (i.e, assuming $[\boldsymbol{\theta}] = [\boldsymbol{\alpha}][\boldsymbol{\beta}][\sigma_{a_0}][\sigma_{b_0}][\sigma_{b_1}][\phi]$), we constructed a Gibbs sampler based on the following full-conditional distributions:

1. The full conditional of b_{0i} depends on the assumed structure of the correlation matrix $\boldsymbol{R}$. Without loss of generality, we may assume

$$[\boldsymbol{b}_0|\beta_0, \sigma_{b_0}, \phi] = \prod_{g=1}^{G} \text{Normal}(\boldsymbol{b}_{0g}|\beta_0 \mathbf{1}, \sigma_{b_0}^2 \boldsymbol{R}_g).$$

where $\boldsymbol{b}_{0g}$ is the subvector of $\boldsymbol{b}_0$ that includes species of guild g. Given this assumption, the model in which abundances are correlated among *all* species can be specified by letting $G = 1$. Likewise, the submodel in which abundances are *uncorrelated* can be specified by assuming $G = I$ and excluding ϕ from the model.

There are two cases to be distinguished in computing the full conditional of b_{0i}:

(a) Guild g has only one species. In this case the full conditional is

$$[b_{0i}|\cdot] = C \exp\{-(b_{0i} - \beta_0)^2/(2\sigma_{b_0}^2)\} \prod_{k=1}^{K} \text{Poisson}(n_{ik}|\lambda_{ik})$$

(b) Guild g has more than one species. In this case the full conditional for species i depends on elements of $\boldsymbol{b}_{0g}$, which includes the related species of i (i.e., species in guild g). Let $\boldsymbol{b}_{0g}^{(-i)}$ denote a subvector of $\boldsymbol{b}_{0g}$ that excludes b_{i0}. Similarly, let $\boldsymbol{R}_g^{(-i)}$ denote the submatrix of $\boldsymbol{R}_g$ obtained by excluding the row and column corresponding to species i. In this case the full conditional is

$$[b_{0i}|\cdot] = C\ \text{Normal}(b_{0i}|\mu_i, \tau_i^2) \prod_{i \in g} \prod_{k=1}^{K} \text{Poisson}(n_{ik}|\lambda_{ik})$$

[1] In this appendix, we use C generically to denote the normalizing constant of a distribution.

where

$$\mu_i = \beta_0 + \sigma_{b_0}^2 \boldsymbol{r}_g^{(i)\prime}(\sigma_{b_0}^2 \boldsymbol{R}_g^{(-i)})^{-1}(\boldsymbol{b}_{0g}^{(-i)} - \beta_0 \boldsymbol{1})$$
$$\tau_i^2 = \sigma_{b_0}^2 \{1 - \sigma_{b_0}^2 \boldsymbol{r}_g^{(i)\prime}(\sigma_{b_0}^2 \boldsymbol{R}_g^{(-i)})^{-1} \boldsymbol{r}_g^{(i)}\}$$

and $\boldsymbol{r}_g^{(i)}$ denotes the ith column of correlation matrix $\boldsymbol{R}_g$.

To sample the full conditional of b_{0i}, we used a random-walk Metropolis sampler with the full conditional density of b_{0i} as target and with a normal proposal distribution. The variance parameter of this proposal was tuned adaptively by incrementing or decrementing the proposal variance depending on whether or not the acceptance rate in each batch of 50 iterations of the Gibbs sampler exceeded a target rate of 0.44 [2] (see Section 4.3.3). We reduced the absolute value of these adjustments in proportion to the inverse square root of the number of batches to ensure that the diminishing-adaptation condition required for convergence (in distribution) of the Markov chain was satisfied [3].

2. We assumed a Normal$(\mu_{\beta_0}, \sigma_{\beta_0}^2)$ prior distribution for β_0 using $\mu_{\beta_0} = 0$ and $\sigma_{\beta_0}^2 = 100$ to convey prior ignorance about this parameter. We used a random-walk Metropolis sampler with the following full conditional density as target

$$[\beta_0|\cdot] = C\ [\beta_0] \prod_{g=1}^{G} \text{Normal}(\boldsymbol{b}_{0g}|\beta_0 \boldsymbol{1}, \sigma_{b_0}^2 \boldsymbol{R}_g)$$

and with a normal proposal distribution. The variance parameter of the proposal was tuned adaptively as described earlier.

3. We assumed a Normal$(\mu_{\beta_1}, \sigma_{\beta_1}^2)$ prior distribution for β_1 using $\mu_{\beta_1} = 0$ and $\sigma_{\beta_1}^2 = 100$ to convey prior ignorance about this parameter. Its full conditional distribution is Normal$((\mu_{\beta_1}/\sigma_{\beta_1}^2 + I\bar{b}_1/\sigma_{b_1}^2)/\tau_{\beta_1},\ 1/\tau_{\beta_1})$, where $\bar{b}_1 = (1/I)\sum_{i=1}^{I} b_{i1}$ and $\tau_{\beta_1} = 1/\sigma_{\beta_1}^2 + I/\sigma_{b_1}^2$

4. For each of the heterogeneity parameters (σ_{b_0}, σ_{b_1}, and σ_{a_0}), we assumed a half-Cauchy prior [4] with unit scale parameter. [4] showed that this prior avoids problems that can occur when alternative "noninformative" priors are used (including the nearly improper, Inverse-Gamma(ϵ, ϵ) family). We estimated each heterogeneity parameter on the log scale (i.e., $\xi = \log(\sigma)$). Therefore, the prior density of each parameter is $[\xi] = 2\exp(\xi)/[\pi\{1 + \exp(2\xi)\}]$. The full-conditional densities of the heterogeneity parameters are:

$$[\xi_{b_0}|\cdot] = C\ [\xi_{b_0}] \prod_{g=1}^{G} \text{Normal}(\boldsymbol{b}_{0g}|\beta_0 \boldsymbol{1}, \sigma_{b_0}^2 \boldsymbol{R}_g)$$

$$[\xi_{b_1}|\cdot] = C\ [\xi_{b_1}] \prod_{i=1}^{I} \text{Normal}(\boldsymbol{b}_{1i}|\beta_1, \sigma_{b_1}^2)$$

$$[\xi_{a_0}|\cdot] = C\ [\xi_{a_0}] \prod_{i=1}^{I} \text{Normal}(\boldsymbol{a}_{0i}|\alpha_0, \sigma_{a_0}^2)$$

To sample each of these full conditionals, we used a random-walk Metropolis sampler with each full conditional density as target and with a normal proposal distribution. The variance parameters of the proposals were tuned adaptively as described earlier.

5. We assumed a half-Cauchy prior with unit scale parameter to convey prior ignorance about ϕ. We estimated this parameter on the log scale (i.e., $\gamma = \log(\phi)$); therefore, the prior density is $[\gamma] = 2\exp(\gamma)/[\pi\{1+\exp(2\gamma)\}]$. The full-conditional density is

$$[\gamma|\cdot] = C\ [\gamma] \prod_{g=1}^{G} \text{Normal}(\boldsymbol{b}_{0g}|\beta_0\mathbf{1}, \sigma^2_{b_0}\boldsymbol{R}_g)$$

To sample this full conditional, we used a random-walk Metropolis sampler with the full conditional density as target and with a normal proposal distribution. The variance parameter of the proposal was tuned adaptively as described earlier.

6. The full conditional of b_{1i} is

$$[b_{1i}|\cdot] = C\exp\{-(b_{1i}-\beta_1)^2/(2\sigma^2_{b_1})\} \prod_{k=1}^{K} \text{Poisson}(n_{ik}|\lambda_{ik})$$

To sample this full conditional, we used a random-walk Metropolis sampler with the full conditional density as target and with a normal proposal distribution. The variance parameter of the proposal was tuned adaptively as described earlier.

7. The full conditional of a_{0i} is

$$[a_{0i}|\cdot] = C\exp\{-(a_{0i}-\alpha_0)^2/(2\sigma^2_{a_0})\} \prod_{k=1}^{K} p_i^{y_{ik\cdot}}(1-p_i)^{J_k n_{ik}-y_{ik\cdot}}$$

where $y_{ik\cdot} = \sum_{j=1}^{J_k} y_{ikj}$ denotes the total number of individuals of species i detected during J_k surveys at location k and where $\text{logit}(p_i) = a_{0i} + \alpha_1 w_{1i}$. To sample this full conditional, we used a random-walk Metropolis sampler with the full conditional density as target and with a normal proposal distribution. The variance parameter of the proposal was tuned adaptively as described earlier.

8. For each of the logit-scale parameters (α_0 and α_1), we used a t-distribution with scale parameter $\sigma = 1.566$ and degrees of freedom parameter $\nu = 7.763$ to specify prior ignorance about these parameters. As shown by [5], this distribution approximates a Uniform$(0,1)$ prior on the inverse-logit scale and assigns low probabilities to values outside the interval (-5,5). Its density function (for generic parameter α) is

$$[\alpha] = \frac{\Gamma((\nu+1)/2)}{\Gamma(\nu/2)\,\sigma\sqrt{(\nu\pi)}}\left(1+\frac{\alpha^2}{\nu\sigma^2}\right)^{-(\nu+1)/2}$$

The full conditionals of the logit-scale parameters are

$$[\alpha_0|\cdot] = C[\alpha_0]\exp\left[-\frac{1}{2\sigma^2_{a_0}}\left\{\sum_{i=1}^{I}(a_{0i}-\alpha_0)^2\right\}\right]$$

and

$$[\alpha_1|\cdot] = C[\alpha_1]\prod_{i=1}^{I}\prod_{k=1}^{K} p_i^{y_{ik\cdot}}(1-p_i)^{J_k n_{ik}-y_{ik\cdot}}$$

To sample each of these full conditionals, we used a random-walk Metropolis sampler with each full conditional density as target and with a normal proposal distribution. The variance parameters of the proposals were tuned adaptively as described earlier.

9. The full conditional of N_{ik} depends on the number of point counts observed at location k. If $J_{ik} = 1$, the full conditional has a shifted-Poisson distribution:

$$N_{ik} - y_{ik1}|\cdot \sim \text{Poisson}(\lambda_{ik}(1 - p_i))$$

If $J_{ik} > 0$, there are two cases to be considered:

(a) No individuals were detected (i.e., $y_{ik\cdot} = 0$). In this case the full conditional is Poisson($\lambda_{ik}(1 - p_i)^{J_k}$).

(b) At least one individual was detected (i.e., $y_{ik\cdot} > 0$). In this case the full conditional is

$$[n_{ik}|\cdot] = \frac{C}{n_{ik}!} \left\{\lambda_{ik}(1 - p_i)^{J_k}\right\}^{n_{ik}} \prod_{j=1}^{J_k} \frac{n_{ik}!}{(n_{ik} - y_{ikj})!}$$

for $n_{ik} \geq max(\boldsymbol{y}_{ik})$. To sample this full condtional, we used a Metropolis-Hastings sampler based on the following shifted-Poisson proposal distribution:

$$N_{ik} - max(\boldsymbol{y}_{ik})|\cdot \sim \text{Poisson}(\lambda_{ik}(1 - p_i))$$

This proposal may be viewed as the full conditional that would have been obtained if a single point count had been observed and its value had equaled the maximum of the J_k point counts actually observed.

We used the Gibbs sampling algorithm described above to construct a Markov chain of 100,000 draws for each model fitted to our data. We discarded the first 50,000 draws as burnin-in and used the remaining $M = 50,000$ draws to estimate posterior means and quantiles of the model parameters and other ecologically relevant functionals of the Markov chain. Monte Carlo standard errors of posterior means and quantiles were computed using the subsampling bootstrap method [6, 7] with overlapping batch means of size $\lfloor\sqrt{M}\rfloor$.

References

1. Geyer CJ (2011) Introduction to Markov chain Monte Carlo. In: Brooks S, Gelman A, Jones GL, Meng XL, editors, Handbook of Markov chain Monte Carlo, Boca Raton, Florida: Chapman & Hall / CRC. pp. 3–48.

2. Rosenthal JS (2011) Optimal proposal distributions and adaptive MCMC. In: Brooks S, Gelman A, Jones GL, Meng XL, editors, Handbook of Markov chain Monte Carlo, Boca Raton, Florida: Chapman & Hall / CRC. pp. 93–111.

3. Roberts GO, Rosenthal JS (2007) Coupling and ergodicity of adaptive Markov chain Monte Carlo algorithms. Journal of Applied Probability 44: 458–475.

4. Gelman A (2006) Prior distributions for variance parameters in hierarchical models (Comment on article by Browne and Draper). Bayesian Analysis 1: 515–534.

5. Dorazio RM, Gotelli NJ, Ellison AM (2011) Modern methods of estimating biodiversity from presence-absence surveys. In: Grillo O, Venora G, editors, Biodiversity Loss in a Changing Planet, Rijeka, Croatia: InTech. pp. 277–302.

6. Flegal JM, Jones GL (2010) Batch means and spectral variance estimators in Markov chain Monte Carlo. Annals of Statistics 38: 1034–1070.

7. Flegal JM, Jones GL (2011) Implementing MCMC: estimating with confidence. In: Brooks S, Gelman A, Jones GL, Meng XL, editors, Handbook of Markov chain Monte Carlo, Boca Raton, Florida: Chapman & Hall / CRC. pp. 175–197.
