# Supplementary material for: Estimating Abundances of Interacting Species Using Morphological Traits, Foraging Guilds, and Habitat
Source: PLoS One. 2014 Apr 11;9(4):e94323. doi: 10.1371/journal.pone.0094323 (PMC3984154; doi:10.1371/journal.pone.0094323)
Supplement: Appendix S3 — Morphological measurements, foraging guild, and sound power output of avian species. (PDF) [file pone.0094323.s003.pdf]

Estimating Abundances of Interacting Species Using Morphological Traits, Foraging Guilds, and Habitat

Robert M. Dorazio[1,*], Edward F. Connor[2]
1 U.S. Geological Survey, Southeast Ecological Science Center, Gainesville, Florida, USA
2 Department of Biology, San Francisco State University, San Francisco, California, USA
∗ E-mail: bdorazio@usgs.gov

Appendix S3: Morphological measurements, foraging guild, and sound power output of avian species.

Table 1: Foraging guild code, body mass (g), morphological measurements (mm), and sound power output (mWatt) of each avian species.

Species	Guild code	Body mass	Beak length	Tarsus length	Wing length	Tail length	Sound power
Wood Duck	1	694	35	36	228	115	71.29
Red-Shouldered Hawk	2	543.5	20.9	82.5	318	200	47.28
Broad-winged Hawk	2	357	18	62	263	159	41.09
Ruffed Grouse	3	621	26.9	43.6	182.4	154.1	64.18
Northern Quail	3	178	14.5	31.7	108.9	60.5	15.44
Mourning Dove	1	130	13.7	21.4	145.4	140	10.13
Yellow-Billed Cuckoo	4	58.8	18.97	25.05	144.8	141.9	4.90
Black-billed Cuckoo	4	48.5	16.84	23.59	137.7	151.4	3.80
Barred Owl	2	630	25.1	63.8	327.8	205.5	65.24
Northern Flicker	6	139.6	30.4	31.1	169	112	11.27
Red-Bellied Woodpecker	8	67.2	30.55	19.1	130.5	78.5	5.09
Hairy Woodpecker	8	73	33.71	21.55	122.14	77.24	5.33
Downy Woodpecker	8	26.6	16.37	16.28	93.67	57.09	1.80
Eastern Kingbird	9	38	14	18.7	117.1	87.8	3.11
Great-crested Flycatcher	9	33.5	15.08	8.41	103.8	91.4	2.30
Eastern Phoebe	9	19.8	14.5	18.1	87.5	72.5	1.26
Acadian Flycatcher	9	13.2	11.2	15.5	75.7	62.5	0.78
Eastern Wood-Peewee	9	14.1	12	13.7	84.4	65.7	0.86
Blue Jay	3	92.4	18.39	34.59	121	121.4	6.81
American Crow	3	458	37.7	57.6	318	180.5	45.36
Black-capped Chickadee	4	10.8	8.75	16.5	65.5	60.5	0.63
Tufted Titmouse	4	21.6	12.1	20.4	79.8	68.5	1.39
White-breasted Nuthatch	8	21.1	19.1	18.6	90.6	47.3	1.36
Red-breasted Nuthatch	8	11	14.1	16.1	68.1	37.1	0.57
Brown Creeper	8	8.4	13.9	15.1	65.5	63.7	0.48
House Wren	4	10.9	12	17	52.5	42.1	0.64
Carolina Wren	4	21.5	12.2	22.3	59.3	49.2	1.18
Northern Mockingbird	3	51.8	17.9	32.5	111.4	119.9	3.51
Gray Catbird	10	36.9	17.5	27.8	92.1	93.5	2.57
Brown Thrasher	10	69	24.9	34.5	103.6	122.5	5.23
American Robin	10	77.3	22.4	33.6	124.7	101.2	5.97
Wood Thrush	3	47.8	11.2	31.6	107.3	70.6	3.42
Hermit Thrush	6	30.1	14.1	30.4	89	64.5	2.11

Continued on next page

Species	Guild code	Body mass	Beak length	Tarsus length	Wing length	Tail length	Sound power
Veery	10	31.9	12.4	29.2	96.4	73.7	2.12
Blue-Gray Gnatcatcher	4	6	9.9	8.9	52.1	50.3	0.32
European Starling	3	83.8	21.34	27.73	130.2	64.78	6.62
White-eyed Vireo	4	11.5	10	19.2	61.3	48	0.67
Yellow-throated Vireo	11	18	11.2	16.9	75.8	48.9	1.13
Red-eyed Vireo	11	20.7	9.5	18.6	80	52.1	1.04
Black-and-White Warbler	8	12	12.2	17.3	68.8	48.5	0.65
Worm-eating Warbler	6	13.2	13	18.4	71.4	53.975	0.78
Blue-winged Warbler	4	11.9	8.7	17.8	60.8	50.9	0.71
Yellow Warbler	4	10	8.02	19.2	64.2	45.1	0.57
Black-throated Green Warbler	11	9.4	12.68	17.3	63.8	47.8	0.50
Cerulean Warbler	11	9.28	9.62	15.7	64.5	42.4	0.55
Chestnut-sided Warbler	4	9.8	9.9	18.3	63.2	50.3	0.57
Prairie Warbler	4	8.2	10.1	18.2	56.2	46.7	0.45
Ovenbird	6	22.5	9.125	21.9	77.7	54.1	1.23
Common Yellowthroat	4	10.3	10.6	20.8	55	49	0.60
Hooded Warbler	4	11.2	10	22.6	68	56.2	0.63
Canada Warbler	4	10.3	10.7	18.7	63.5	53.5	0.62
American Redstart	4	8.6	9.11	17.9	63.5	55.1	0.48
Red-winged Blackbird	13	70.5	23.6	30.2	120.9	91.7	4.78
Baltimore Oriole	14	35.2	13	22.7	95.1	76.6	2.36
Common Grackle	3	131.4	30.1	34.4	139.3	141.4	10.51
Brown-headed- Cowbird	13	48.9	17.3	25	110.2	77	3.55
Scarlet Tanager	11	24.5	10.4	19.8	94.5	67.5	1.92
Northern Cardinal	3	45.1	12.5	24.2	95.2	96	3.25
Rose-breasted Grosbeak	14	45.6	16.9	22.5	104.4	74.6	3.27
American Goldfinch	10	13.2	10.5	14.4	71.1	48.1	0.80
Eastern Towhee	3	41.7	14.3	26.5	85.8	91	2.95
Chipping Sparrow	3	13.25	9.3	17.3	72	62.3	0.73
Field Sparrow	3	13.1	10.5	17.1	66.1	62.1	0.75
Song Sparrow	10	25	15.87	24.37	71.4	69.86	1.35
Red-tailed Hawk	2	1028	25.1	82.4	370.1	215.4	114.00
Cedar Waxwing	9	30.8	6.76	16.4	94.7	52.7	2.07
Eastern Bluebird	6	29.5	10	22.6	100	65	2.15
Whip-poor-will	5	50.3	6.5	22	156.7	120	3.96
Pileated Woodpecker	7	305	50	34.5	235	171	28.85
Louisiana Waterthrush	12	20.4	13.2	22.3	80.8	51.8	1.26
House Finch	15	21.5	8.2	17.23	80.55	67.1	1.38
Fish Crow	16	299.9	30.7	46.5	284.9	156	28.00
Ruby-throated Hummingbird	17	3	16.4	5.05	38.8	27.6	0.18

Table 2: Codes and descriptions of foraging guilds.

Guild code	Guild description
1	granivore: ground gleaner and freshwater surface gleaner
2	carnivore: ground hawker
3	omnivore: ground forager
4	insectivore: lower canopy gleaner
5	insectivore: air screener
6	insectivore: ground gleaner
7	insectivore: bark excavator
8	insectivore: bark gleaner
9	insectivore: air sallier
10	omnivore: ground and lower canopy forager
11	insectivore: upper-canopy gleaner
12	insectivore: freshwater shoreline gleaner
13	granivore: ground forager
14	omnivore: upper canopy forager
15	frugivore: ground gleaner
16	omnivore: shorline scavenger
17	omnivore: floral hover-gleaner
