# Supplementary material for: Estimating Abundances of Interacting Species Using Morphological Traits, Foraging Guilds, and Habitat
Source: PLoS One. 2014 Apr 11;9(4):e94323. doi: 10.1371/journal.pone.0094323 (PMC3984154; doi:10.1371/journal.pone.0094323)
Supplement: Appendix S4 — Estimates of model parameters for avian point-count data. (PDF) [file pone.0094323.s004.pdf]

Estimating Abundances of Interacting Species Using Morphological Traits, Foraging Guilds, and Habitat

Robert M. Dorazio[1,*], Edward F. Connor[2]
1 U.S. Geological Survey, Southeast Ecological Science Center, Gainesville, Florida, USA
2 Department of Biology, San Francisco State University, San Francisco, California, USA
∗ E-mail: bdorazio@usgs.gov

Appendix S4: Estimates of model parameters for avian point-count data.

Table 1: Posterior means and standard errors for species-specific abundance parameters: b_0 = logarithm of mean abundance at average value of forested area, b_1 = effect of forested area on mean abundance.

Species	b_0	SE(b_0)	b_1	SE(b_1)
Wood Duck	-1.749	1.01	-0.289	0.544
Red-Shouldered Hawk	-1.702	0.81	0.118	0.428
Broad-winged Hawk	-1.067	0.79	0.188	0.383
Ruffed Grouse	-0.784	0.84	0.893	0.429
Northern Quail	0.088	0.50	-0.789	0.211
Mourning Dove	-0.711	0.29	0.075	0.190
Yellow-Billed Cuckoo	-0.362	0.42	-0.440	0.205
Black-billed Cuckoo	-1.549	0.58	0.109	0.334
Barred Owl	-1.515	1.04	0.021	0.477
Northern Flicker	0.782	0.42	-0.482	0.126
Red-Bellied Woodpecker	-0.251	0.47	0.894	0.196
Hairy Woodpecker	-0.059	0.42	0.324	0.181
Downy Woodpecker	0.162	0.38	-0.338	0.153
Eastern Kingbird	-2.552	0.78	-0.452	0.481
Great-crested Flycatcher	0.814	0.27	-0.156	0.101
Eastern Phoebe	-1.105	0.47	-0.239	0.270
Acadian Flycatcher	-1.920	0.60	-0.264	0.372
Eastern Wood-Peewee	0.442	0.21	0.386	0.107
Blue Jay	1.850	0.41	-0.158	0.072
American Crow	1.431	0.41	-0.293	0.088
Black-capped Chickadee	1.660	0.36	-0.206	0.079
Tufted Titmouse	1.604	0.31	-0.110	0.070
White-breasted Nuthatch	1.232	0.40	0.271	0.111
Red-breasted Nuthatch	-2.894	0.88	-0.041	0.501
Brown Creeper	-0.789	0.45	0.603	0.234
House Wren	-0.175	0.19	-0.770	0.138
Carolina Wren	-1.310	0.44	-1.017	0.262
Northern Mockingbird	-1.329	0.51	-0.768	0.290
Gray Catbird	-0.336	0.23	-0.980	0.160
Brown Thrasher	-2.234	0.66	-0.542	0.415
American Robin	-0.012	0.30	-0.169	0.156
Wood Thrush	1.232	0.19	0.305	0.074
Hermit Thrush	-2.033	0.65	0.974	0.378
Veery	0.713	0.13	0.047	0.084

Continued on next page

Species	b_0	SE(b_0)	b_1	SE(b_1)
Blue-Gray Gnatcatcher	-1.059	0.67	1.014	0.304
European Starling	-1.397	0.53	-1.192	0.305
White-eyed Vireo	-2.199	0.55	-1.645	0.363
Yellow-throated Vireo	-1.800	0.59	1.274	0.347
Red-eyed Vireo	1.245	0.17	0.396	0.072
Black-and-White Warbler	0.357	0.19	-0.066	0.111
Worm-eating Warbler	-0.074	0.29	0.597	0.148
Blue-winged Warbler	-0.765	0.48	-0.614	0.243
Yellow Warbler	-1.899	0.58	-1.422	0.374
Black-throated Green Warbler	-2.319	0.43	0.399	0.329
Cerulean Warbler	-2.215	0.69	0.912	0.409
Chestnut-sided Warbler	-2.074	0.62	-0.717	0.380
Prairie Warbler	-2.018	0.36	-0.361	0.299
Ovenbird	1.518	0.15	0.491	0.061
Common Yellowthroat	0.062	0.19	-0.844	0.126
Hooded Warbler	-0.901	0.27	0.418	0.196
Canada Warbler	-1.647	0.56	0.213	0.342
American Redstart	-0.840	0.41	0.390	0.224
Red-winged Blackbird	-1.163	0.35	-0.962	0.238
Baltimore Oriole	0.669	0.37	-0.089	0.135
Common Grackle	-0.045	0.47	-0.758	0.201
Brown-headed- Cowbird	0.933	0.40	0.084	0.122
Scarlet Tanager	1.164	0.33	0.353	0.090
Northern Cardinal	0.624	0.25	-0.529	0.107
Rose-breasted Grosbeak	-0.189	0.54	0.427	0.232
American Goldfinch	-1.411	0.66	-0.591	0.354
Eastern Towhee	1.036	0.13	-0.273	0.075
Chipping Sparrow	-2.749	0.89	-0.193	0.503
Field Sparrow	-2.868	0.83	0.060	0.502
Song Sparrow	-2.082	0.35	-1.085	0.293
Red-tailed Hawk	-1.084	1.05	0.340	0.554
Cedar Waxwing	-2.741	0.87	-0.129	0.496
Eastern Bluebird	-2.462	0.73	0.049	0.461
Whip-poor-will	-3.089	0.85	-0.199	0.501
Pileated Woodpecker	-1.275	0.71	0.678	0.387
Louisiana Waterthrush	-1.260	0.67	0.772	0.333
House Finch	-1.777	0.70	-0.802	0.411
Fish Crow	-1.779	0.78	-0.961	0.465
Ruby-throated Hummingbird	-2.884	0.88	-0.066	0.512
